# Clinical phase I/II trial of SVF therapy for cartilage regeneration: A cellular therapy with novel 3D MRI imaging for evaluating chondral defect of knee osteoarthritis

**DOI:** 10.3389/fcell.2023.1106279

**Published:** 2023-01-19

**Authors:** Bin Ren, Yiqiang Chang, Ruolan Liu, Feng Xiao, Jun Xu, Lingsong Li, Tao Li, Zhao Ruan, Yigui Bao, Junbing Lin, Junying Zhou, Weijing Liao, Zhenyu Pan, Haibo Xu, Jun Tian, Lin Cai, Xin Xiao Zheng

**Affiliations:** ^1^ Department of Orthopedics, Zhongnan Hospital of Wuhan University, Wuhan, China; ^2^ Department of Rehabilitation Medicine, Zhongnan Hospital of Wuhan University, Wuhan, China; ^3^ Department of Radiology, Zhongnan Hospital of Wuhan University, Wuhan, China; ^4^ Plastic Surgery, General Hospital of the People’s Liberation Army, Beijing, China; ^5^ Shanghai Advanced Research Institute, Chinese Academy of Sciences, Shanghai, China; ^6^ Clinic Laboratory, Zhongnan Hospital of Wuhan University, Wuhan, China; ^7^ Department of Plastic Surgery, University of Pittsburgh Medical Center, Pittsburgh, PA, United States; ^8^ Transplantation Medical Center, Zhongnan Hospital of Wuhan University, Wuhan, China

**Keywords:** stromal vascular fraction (SVF), cartilage regeneration, 3D MRI imaging, osteoarthritis, clinical trial

## Abstract

**Background:** The clinical applications of stromal vascular fraction (SVF) therapy for osteoarthritis (OA) have attracted academic and clinical attention. However, data of the effects of stromal vascular fraction therapy on regeneration of degenerated cartilage are limited in the literature. Meanwhile, there is a great need for a simple and non-invasive evaluation method to analyze the changes of joint cartilage qualitatively and quantitatively in clinical trials. This study entitled “stromal vascular fraction Therapy for Human Knee Osteoarthritis” was registered in ClinicalTrial.gov # NCT05019378.

**Materials and Methods:** We designed and conducted a single center, open labeled clinical phase I/II study, and 6 osteoarthritis patients with both knee cartilage defect I-II were enrolled in this study. The two knees of each patient were randomly assigned to autologous stromal vascular fraction treatment group or non-treatment control group to evaluate the safety and therapeutic effect of stromal vascular fraction therapy for human knee osteoarthritis. We have also established a novel protocol to provide 3D MRI imaging for human knee cartilage enabling us to qualitatively and quantitatively evaluate cartilage degeneration and regeneration in this study.

**Results:** The qualitative and quantitative evaluation of 3D Magnetic Resonance Imaging (MRI) imaging of knee cartilage demonstrated that the stromal vascular fraction therapy reduced the cartilage defects; and significant increase of cartilage value both in defect cartilage area and whole cartilage area of treated group and significant increase of thickness and area of both femoral and tibia cartilage in vertical sections of the stromal vascular fraction treated Group at 12 and 24 W post treatment in cartilage defect I-II osteoarthritis patients.

**Conclusion:** This clinical phase I/II study indicated that stromal vascular fraction therapy is a safe clinical procedure and provided evidence that the stromal vascular fraction therapy significantly facilitated cartilage regeneration, opening the opportunity to a phase III trial investigating authentic efficacy of the procedure. This study is the first qualitative and quantitative evaluation of the efficacy of autologous stromal vascular fraction cellular therapy on cartilage regeneration. Through early and definite diagnosis of knee osteoarthritis patients, and providing safe and efficient therapy to facilitate cartilage regeneration, we will be able to control or reverse cartilage degeneration and completely change the epidemiology of osteoarthritis worldwide.

## 1 Introduction

Osteoarthritis (OA) is one of the most common joint disorders and the leading causes of work restrictions and decreased quality of life in the elderly (Dunlop et al., 2003). The cartilage degeneration is commonly considered to be the initial pathological defect in OA. However, current clinical interventions for OA, including knee arthroplasty, are all during the stage of managing symptoms rather than regenerative medicine (Coleman et al., 2010; Gademan et al., 2016; Kizaki et al., 2019). With the aging of the population, osteoarthritis patients are increasing year-by-year and effective cartilage regeneration approaches are urgently needed.

Recently, adult mesenchymal stem cells (MSCs) or adipose-derived stem cells (ADSCs) have emerged as promising candidates with great healing potential in regenerative medicine because of their capacity to differentiate into multiple tissue types and to self-renew (Richardson et al., 2016; Gu et al., 2017; Vural et al., 2017; Bonaventura et al., 2020). Nevertheless, before utilizing stem cells for cartilage regeneration by tissue engineering methods, *in vitro* amplification is necessary to obtain sufficient number of cells before transplantation. However, local microenvironment of culture media can affect stem cells differentiation and *ex vivo* manipulation may result in genetic changes that may affect functional and biological characteristics. Moreover, progenitor cells are able to regenerate damaged articular cartilage, but the lack of vascularity in cartilage region prevents the infiltration and survival of the implanted stem cells. Therefore, recent regenerative medicine switched to apply stromal vascular fraction (SVF) instead of MSCs or ADSCs, aiming to resolve these limitations in tissue engineering.

Stromal vascular fraction (SVF) isolated from adipose tissues enriched adipose stem cells (ADSCs) as well as other stromal cell types involved in tissue regeneration such as endothelial cells, pericytes, and fibroblasts (Charles-de-Sa et al., 2015; You et al., 2015). These various cell components in SVF may act synergistically with stem cells in facilitating and promoting tissue regeneration (Ramakrishnan and Boyd, 2018). More importantly, SVF cells can be readily obtained from liposuction procedure without the need for any cell propagation, which make it more convenient and feasible for clinical application than culture-expanded ADSCs (Pak et al., 2016a; Bora and Majumdar, 2017; Desando et al., 2019). The clinical applications of SVF therapy for osteoarthritis (OA) have attracted academic and clinical attention (Comella et al., 2017; Farre-Guasch et al., 2018; Tran et al., 2019; Bonaventura et al., 2020). On the ClinicalTrials.gov website, 34 clinical observations utilizing SVF have been registered, of which 7 are for the treatment of osteoarthritis. In the limited published clinical observations, visual analog score (VAS) for pain, functional rating index (FRI), range of motion (ROM) of the patients, and the Western Ontario and McMaster Universities Arthritis Index (WOMAC) questionnaire were assessed to evaluate the outcome of the treatment (Koh et al., 2014; Koh et al., 2015; Fodor and Paulseth, 2016). However, these measurement methods do not provide direct evidences of cartilage degeneration or regeneration.

Conventional Magnetic Resonance Imaging (MRI) can be performed to assess the extent of cartilage damage (Baysal et al., 2004), but cannot be analyzed quantitatively. In addition, most studies utilize two-dimensional MRI images to compare the changes in cartilage between different time periods of the same patient. However, there is no reliable criterion or protocol to ensure that the two images of the MRI section of the same patients from different time periods are actually anatomically identical. At present, there is a great need for a simple and non-invasive evaluation method to analyze the changes of joint cartilage qualitatively and quantitatively in clinical trials.

Herein we reported a randomized clinical phase I/II trial to evaluate the safety and therapeutic effect of autologous SVF cellular therapy on knee OA. In addition, a novel method to provide 3-dimension (3D) MRI imaging for human knee cartilages and bones was developed, enabling us to qualitatively and quantitatively evaluate cartilage regeneration in this study.

## 2 Materials and methods

Study participants voluntarily provided written informed consent to participate in the study and signed the Health Insurance Portability and Accountability Act (HIPAA) authorization letter before participating in any research procedures. The study was reviewed and approved by the Medical Ethical Committee of Zhongnan Hospital of Wuhan University and was conducted in accordance with the Declaration of Helsinki, International Conference on Harmonization (ICH).

### 2.1 Study design and participants

We designed and conducted a single center, open labeled clinical phase I/II study. A total of 6 OA patients were enrolled in this study based on their clinical and MRI evaluation. Inclusion criteria were men and women, aged 35–70 years, both knee joints with articular cartilage defect grades I/II, body mass index (BMI) less than 35 kg/m^2^. Patients meeting the following criteria were excluded: serious medical disorders, previous major knee trauma, mechanical pain caused by severe meniscus injury, autoimmune or inflammatory arthritis, intra-articular hyaluronic acid or corticosteroid injection in the preceding 3 months, previous arthroscopic or open surgery treatment for knee OA in the past 6 months, and inability to provide informed consent. The two knees of each patient were randomly assigned to an autologous SVF (10^8^ cells) treatment group or non-treatment control group. The patients were evaluated every 4 weeks up to 24 weeks for safety and efficacy of autologous SVF therapy (Table 1).

**TABLE 1:** The clinical trial design of the SVF cellular therapy with novel 3D MRI imaging for OA.

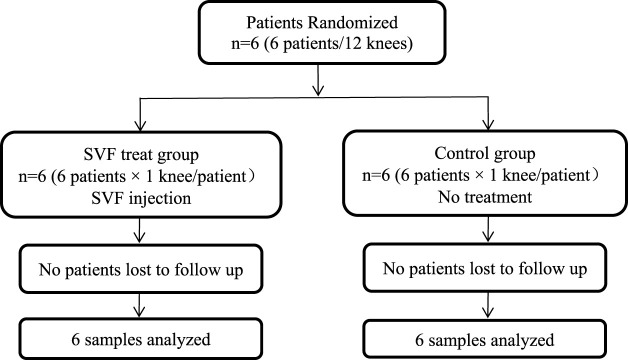

### 2.2 Adipose tissue harvest

The adipose tissue harvest and subsequent SVF cells processing protocols were followed within the guidelines of the International Federation for Adipose Therapeutics and Science (IFATS) and the International Society for Cellular Therapy (ISCT) (Bourin et al., 2013; Bora and Majumdar, 2017). On the day of SVF injection, subcutaneous adipose tissue was harvested from the patients’ abdomen by liposuction with tumescent technique (Stone, 1995). Through a small incision, a 3.7 mm hollow blunt-tipped cannula was inserted into the abodominal subcutaneous tissue of the subject, and tumescent solution (0.9% saline solution supplemented with 2% lidocaine, 8.4% sodium hydrogen carbonate, and 0.1% epinephrine) was administered into adipose tissue through infiltration before aspiration. Liposuction with a target volume of about 200–250 mL was collected directly into a sterile tissue-processing container to produce SVF. After the liposuction procedure, pressure bandages were applied around the abdomen for 48 h.

### 2.3 Preparation of SVF cells

The adipose tissue was then transported to the certified cell therapy laboratory. The adipose tissue was washed twice in PBS and digested through mixing with collagenase Type I (Washington Biochemical Corp., NJ, USA) at 37°C for 30 min with agitation at 5-min intervals. Afterwards, PBS-EDTA was added to stop collagenase activity. The solution was then centrifuged at 800 g for 10 min to collect the cell pellet. The supernatant, including all debris, floating cells and the aqueous phase, was removed and discarded. The pellet was resuspended with normal saline 0.9% to obtain 3 mL of cell solution containing 10^8^ SVF cells for injection into each knee joint. Prior to injection, a cell viability test, endotoxin test, and bacteriologic tests were carried out before releasing for injection. In addition, an aliquot sample was sent to a third-party cetified laboratory for quality control tests.

### 2.4 SVF treatment

The patient was kept in a supine position on the operating table. Before the treatment, local anesthesia in the knee joint was administered. The Ultrasound-guided intro knee joint injection was performed in the operation room. Three milliliters of cell suspension containing 10^8^ SVF cells were slowly injected into the joint through an 18 gauge 1.5-inch needle. The injection site was disinfected with povidone-iodine solution and covered with a sterile bandage. After injection, the subject’s knee was kept in zero load state for 48 h.

### 2.5 Follow-up and clinical assessment

At the baseline, enrolled patients were assessed for vital signs, laboratory tests, knee pain and daily functional mobility, 3D MRI imaging evaluations of articular cartilage defect, volume and thickness. Clinical status of all patients was closely monitored at baseline, at the time of SVF treatment, 12 and 24 weeks after the SVF treatment. Knee pain, functional mobility and physical disability were assessed using the WOMAC patient questionnaire and the Lysholm knee scale. Clinical evaluation including medical history, physical examination, as well as any side effects possibly associated with SVF cell therapy were also carefully documented.

### 2.6 Establishment of a novel method to provide 3D MRI imaging for evaluating human knee cartilage

An experienced orthopedic radiologist, who was blind to the grouping and treatment, performed the comparative evaluation of images before and after SVF therapy.

#### 2.6.1 MRI data acquisition

The scanning sequence of a human knee joint is obtained based on tomography technology. The parameters of the MRI scanner with high resolution Siemens 3.0 T MRI device (Siemens, Munich, Germany) are as follows: repetition time (TR): 14.10 ms; echo time (TE): 5.0 ms; field of view (FOV): 171 mm Å∼ 171 mm; data matrix:320 Å∼ 320; slice thickness: 0.53 mm。By setting the parameters of the scanner to the set value, it is possible to obtain a greater differentiation between various soft tissues (such as articular cartilage, ligament, articular capsule and synovium) and hard bones. When photographing the human knee joint with the tomographic technology, the body position of the photographed person and the degree of flexion and extension of the joint part are fixed.

#### 2.6.2 Establishment of human knee joint MRI 3D models

**FIGURE 1:**
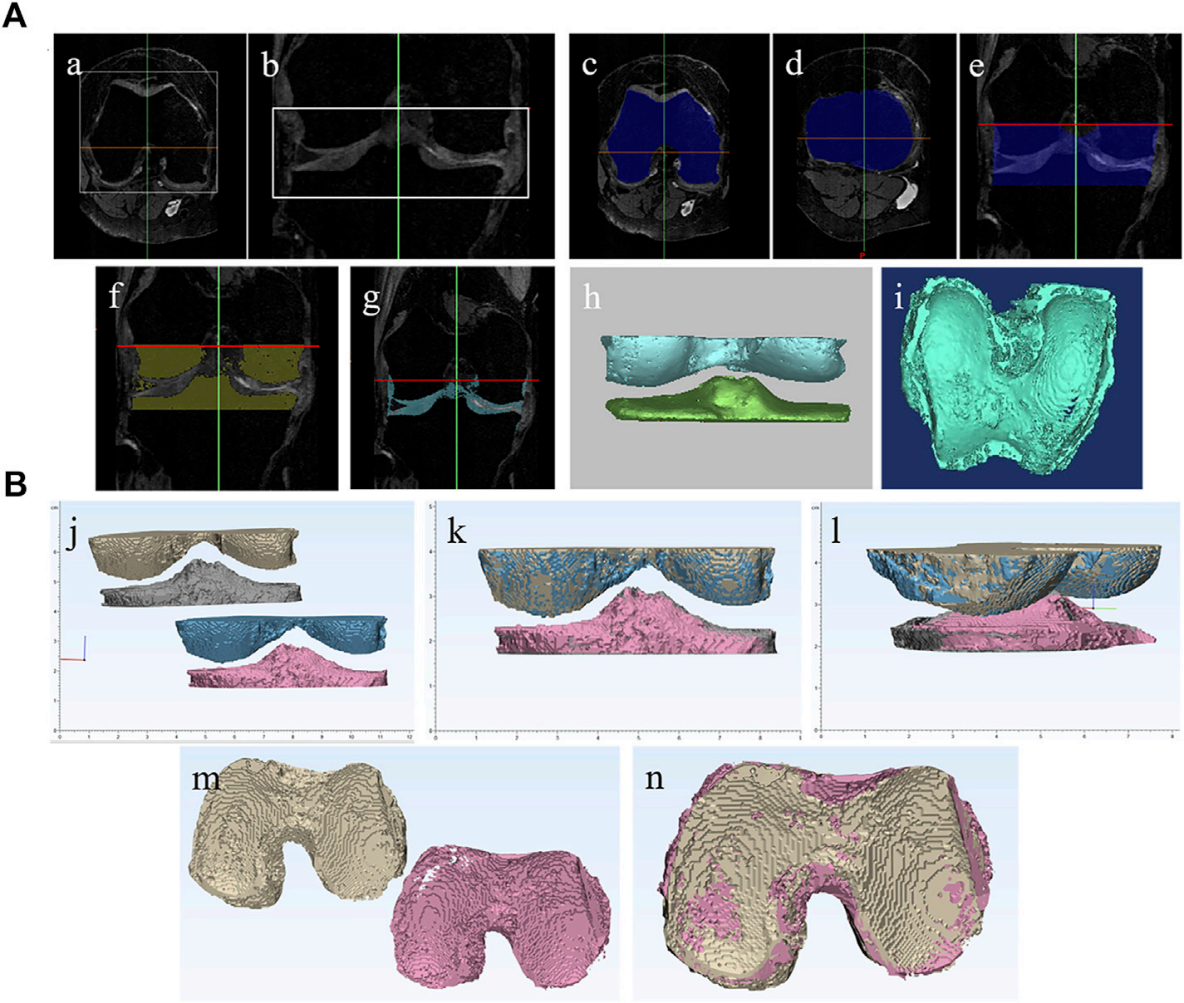
Establishment of human knee joint MRI 3D models and 3D spatial alignment method. **(A)** Establishment of human knee joint MRI 3D models: **(a,b)** The detection range from the femoral condyle to the tibial plateau of knee joint is selected in coronal plane and sagittal plane of MRI; **(c–e)** region growing method is used to obtain the contour curves of all planes of the detection range; **(f,g)** Based on the gray scale thresholds of cartilage or bone, the scanning sequences of cartilage or bone within the selected region are identified respectively. **(h,i)** the cartilage and bone 3D MRI models are generated. **(B)**. Establishment of 3D spatial alignment method: **(j-n)** 3-matic was applied to spatially align the MRI image data I and II based on osteogenic section to ensure that the cartilage section is registered in the same 3D spatial domain.

Acquired DICOM data were processed by Mimics ver. 17.0 (Materialise, Leuven, Belgium) to establish 3D knee joint cartilage imaging models.

a. The detection range from the femoral condyle to the tibial plateau of the knee joint is selected in coronal plane and sagittal plane of the MRI (Figures 1a, b).
b. The contour curves of the tibia and femur of the knee joint are drawn in the top and bottom coronal plane of the detection range. Based on these two contour curves, the region growing method is used to obtain the contour curves of all planes of the detection range (Figures 1c–e).
c. The grayscale threshold value of knee joint bone and cartilage are determined separately. Based on the grayscale thresholds of cartilage or bone, the scanning sequences of cartilage or bone within the selected region are identified respectively (Figures 1f, g). Then the cartilage and bone 3D MRI models are generated (Figures 1h, i).

Within the selected detection range of the knee joint, the grayscale threshold of bone tissue rarely overlaps with that of other tissues. There is no need for special handling after 3D imaging, and only the redundant parts, which are not consistent with the anatomic structure, can be removed.

Within the selected detection range of the knee joint, the grayscale threshold of articular cartilage may overlap with the meniscus and the cruciate ligament to a certain extent. After 3D imaging, targeted processing can be carried out as needed.

#### 2.6.3 Establishment of MRI 3D imaging model for qualitative analysis of articular cartilage defects

**FIGURE 2:**
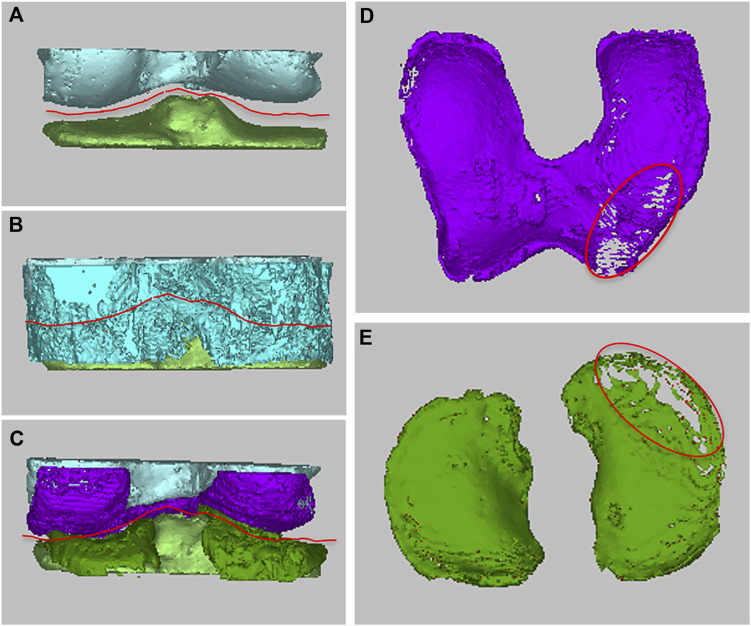
Qualitative analysis of femoral cartilage and tibial cartilage. **(A–C)** The articular cartilage was determined with the articular surface of femur or tibia as the reference template, **(D,E)** the damage of cartilage were qualitatively analyzed based on the defect of articular cartilage.

Since the knee articular cartilage is a thin layer of cartilage attached to the articular surface of the femur and tibia, the articular surface of the femur and tibia can be used as a reference to identify a thin bowl-shaped layer of cartilage. Other tissues with the same grayscale value that are not associated with articular cartilage can be removed by the regional growth method or by manual erase. The purpose of 3D image qualitative analysis of articular cartilage is to determine whether there is cartilage defect and the dynamic change of defect before and after SVF treatment (Figure 2). We have drafted the ZhongnanH articular cartilage defect index for this study: I) Sporadic cartilage defects; II) Single cartilage defect <200 mm^2^ or total defect <600 mm^2^; III) Large single cartilage defect >200 mm^2^ or total defect >600 mm^2^; IV) Penetrating cartilage defect: on the same contact surface, both tibial cartilage and femoral cartilage are defective. Articular cartilage defect grade I has the least impact on the knee joint, while grade IV causes direct bone friction, inducing serious knee dysfunction.

#### 2.6.4 Establishment of 3D spatial alignment method for quantitative analysis of articular cartilage

**FIGURE 3:**
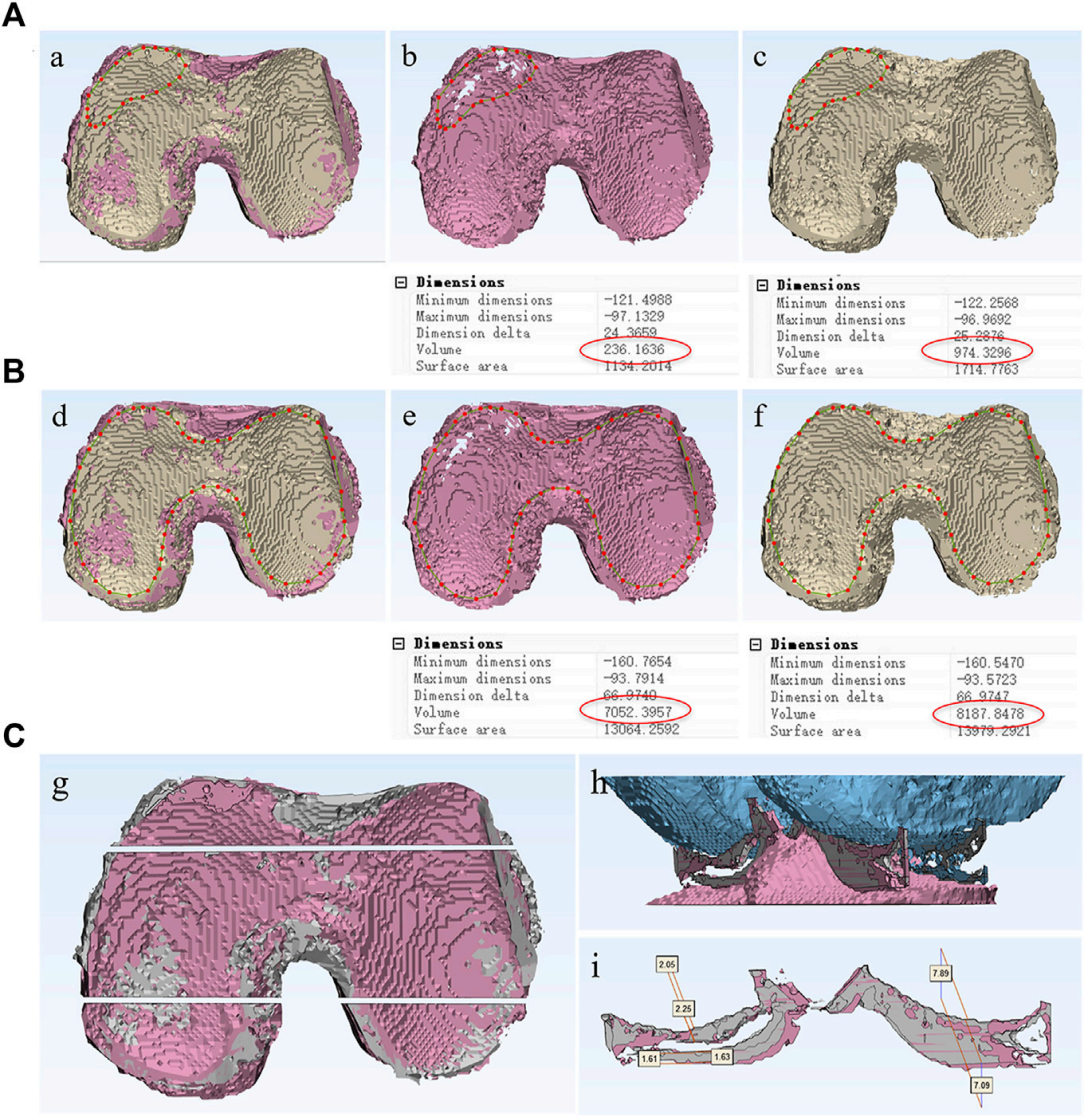
Quantitative analysis method of articular cartilage at different time periods. Define the borderline of defect cartilage area **(A)** or entire articular cartilage area **(B)**, and the volume changes between two different periods could be measured and compared. **(C)** The knee joint was coronal incised at the anterior and posterior positions, the cross-sectional area of cartilage, as well as the thickness of femoral cartilage and tibial cartilage at each position could be measured respectively.

To achieve quantitative comparative analysis of the same patient’s articular cartilage, MRI imaging data between two different periods (refer to MRI imaging data I and II), we spatially align the MRI imaging data I and II based on osteogenic section to ensure that the cartilage section is registered in the same 3D spatial domain (Figure 1B). In this manner, the cartilage volume, area, and thickness of the same patient between two different time periods can be quantitatively compared (Figure 3).

By defining the borderline of the entire articular cartilage area or defect cartilage area, the volume changes between two different periods can be measured and compared (Figures 3A, B). In order to further evaluate the status of cartilage at different positions, the knee joint was coronal incised at the anterior and posterior positions. The cross-sectional area of cartilage in each incision, as well as the thickness of femoral cartilage and tibial cartilage at the anterior and posterior positions was measured respectively (Figure 3C). The thickness and cross-sectional area of cartilage at different time periods, as well as the total volume and defect volume of cartilage, were used to reflect the changes of cartilage state before and after treatment.

### 2.7 Statistical analysis

Statistical analysis was performed using GraphPad Prism v6.0 (GraphPad Software, San Diego, CA, United States). Data were presented as mean ± standard error. The radiologic evaluation including the volume of whole or damaged cartilage, the cross-sectional area of cartilage in each incision, as well as the thickness of femoral cartilage and tibial cartilage were analyzed by two-way analysis of variance (ANOVA) to compare the data at each time-point of the SVF treated group with the corresponding time-point of the control group and to check the significance between the two groups. In addition, for each group the 24 weeks outcome at different time points was compared to check the change of cartilage status. Statistical inferences were based on a paired *t*-test for WOMAC and Lysholm analysis. The Mann-Whitney *U*-test was applied when the assumptions of a *t*-test were not met.

## 3 Result

### 3.1 Patient characteristics

Six patients with bilateral knee OA were enrolled in this clinical phase I/II study, and the unilateral knee joint of each patient was randomly divided into the treatment group [autologous SVF (10^8^ cells) therapy] or the control group (no treatment). The mean age was 62.17 ± 6.34 years (range 53–69 years), 5 (83.3%) patients were females and 1 (16.7%) was male. There were 3 (50.0%) patients with normal weight (BMI 18–24.9), 3 (50.0%) overweight patients (BMI 25–29.9). All the enrolled patients were diagnosed with cartilage defect I-II based on MRI 3D imaging. All patients were evaluated regularly during their 24-week follow-up visits.

### 3.2 SVF cells therapy

Among the enrolled patients, the average amount of harvested liposuction was 200 mL, with an average SVF cells count of 1.5 × 10^8^ cells. The aliquot cells were processed through internal and centisified third party lab quality control protocol for viability, cell count, endotoxin, and Gram-positive bacterial testing. The SVF products from the 6 patients met releasing standards. The cellular components of SVF were characterized by FACS analysis as following: CD45^+^ cells (Blood-derived cells, 40.81 ± 2.73%), CD45-/CD31-/CD34+ cells (Adipose-derived stromal cells, 33.86 ± 3.40%), CD45-/CD31+/CD34+ cells (Vascular endothelial cells, 10.45 ± 1.82%) and CD45-/CD31-/CD34-cells (other cells including Fibroblasts, Pericytes and others, 14.87 ± 2.99%) (Bourin et al., 2013; Bora and Majumdar, 2017). The SVF injections were completed in the operation room. Under local anesthesia with 1% lidocaine and ultrasound-guidance, 2 mL of 10^8^ SVF cell suspension were injected into the medial side of the knee joint. The SVF cells processing time from the end of adipose tissue harvest to prepared SVF cells in syringe for injection was 120–150 min. The aliquot SVF cells were sent to a national certified-laboratory for quality control testing and all reports were consistent with our internal testing results.

### 3.3 Safety evaluation

No complications related to adipose tissue harvest were noticed. There were no other adverse events including infection, thrombo-embolism, poor wound healing, or allergy associated with SVF cell administration. Each patient’s EKG, blood tests (kidney, liver function) remained normal throughout the follow up period.

### 3.4 Qualitative evaluation of cartilage defect of OA patients

**FIGURE 4:**
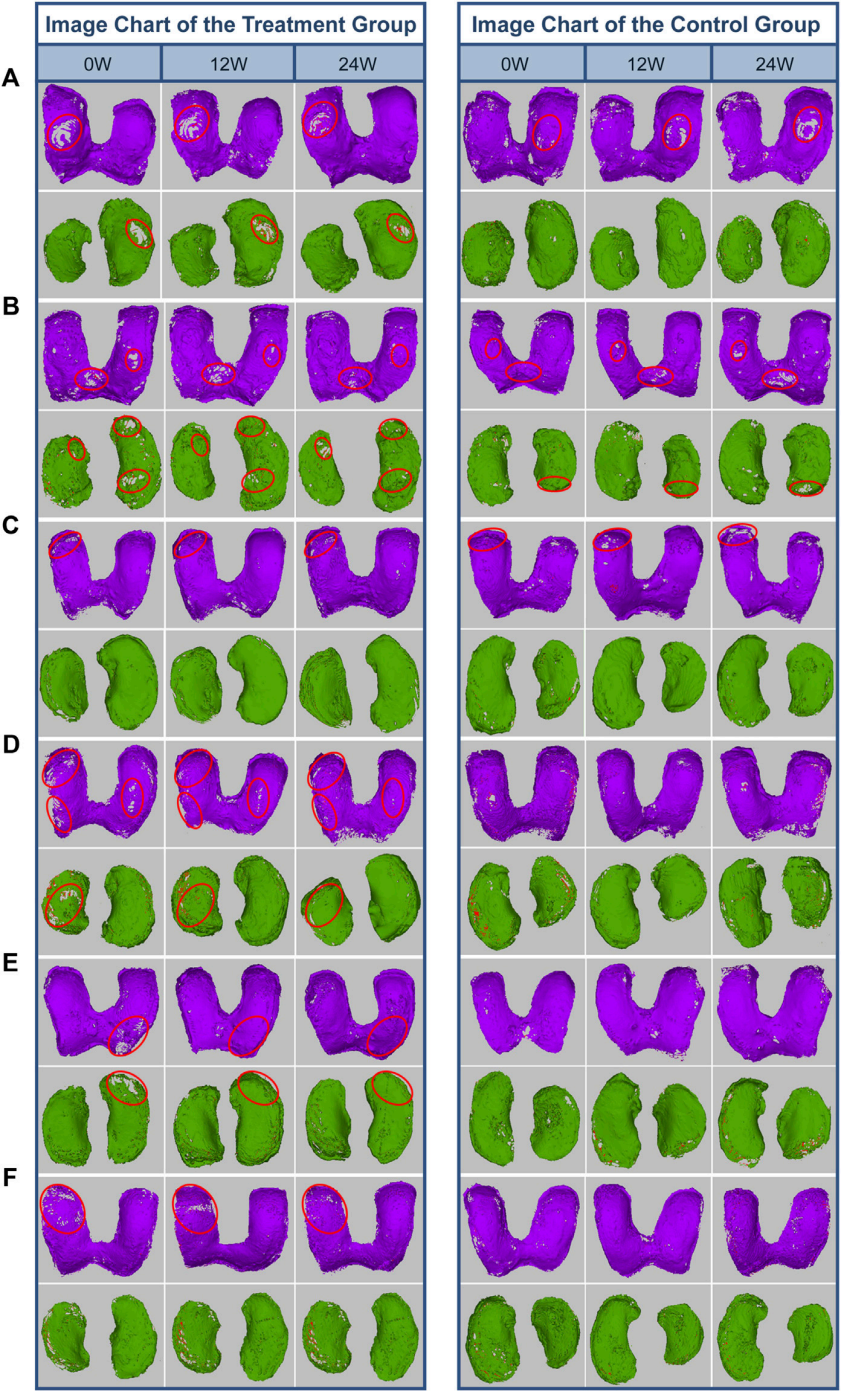
Qualitative analysis of femoral cartilage and tibial cartilage defects of 6 enrolled patients. **(A)** Patient 1: female, 69 years, BMI = 23.5. **(B)** Patient 2: female, 69 years, BMI = 20. **(C)** Patient 3: female, 64 years, BMI = 23.8. **(D)** Patient 4: female, 59 years, BMI = 29.2. **(E)** Patient 5: female, 59 years, BMI = 27.3. **(F)** Patient 6: male, 53 years, BMI = 25.0. After a single injection of SVF the femur and/or tibia cartilage defects of all 6 patients show improvement, some show significant reduction at 12 and 24 weeks post therapy in comparison with pretreatment. In contrast, most the defects of untreated group show deterioration and some new defects are noted.

**FIGURE 5:**
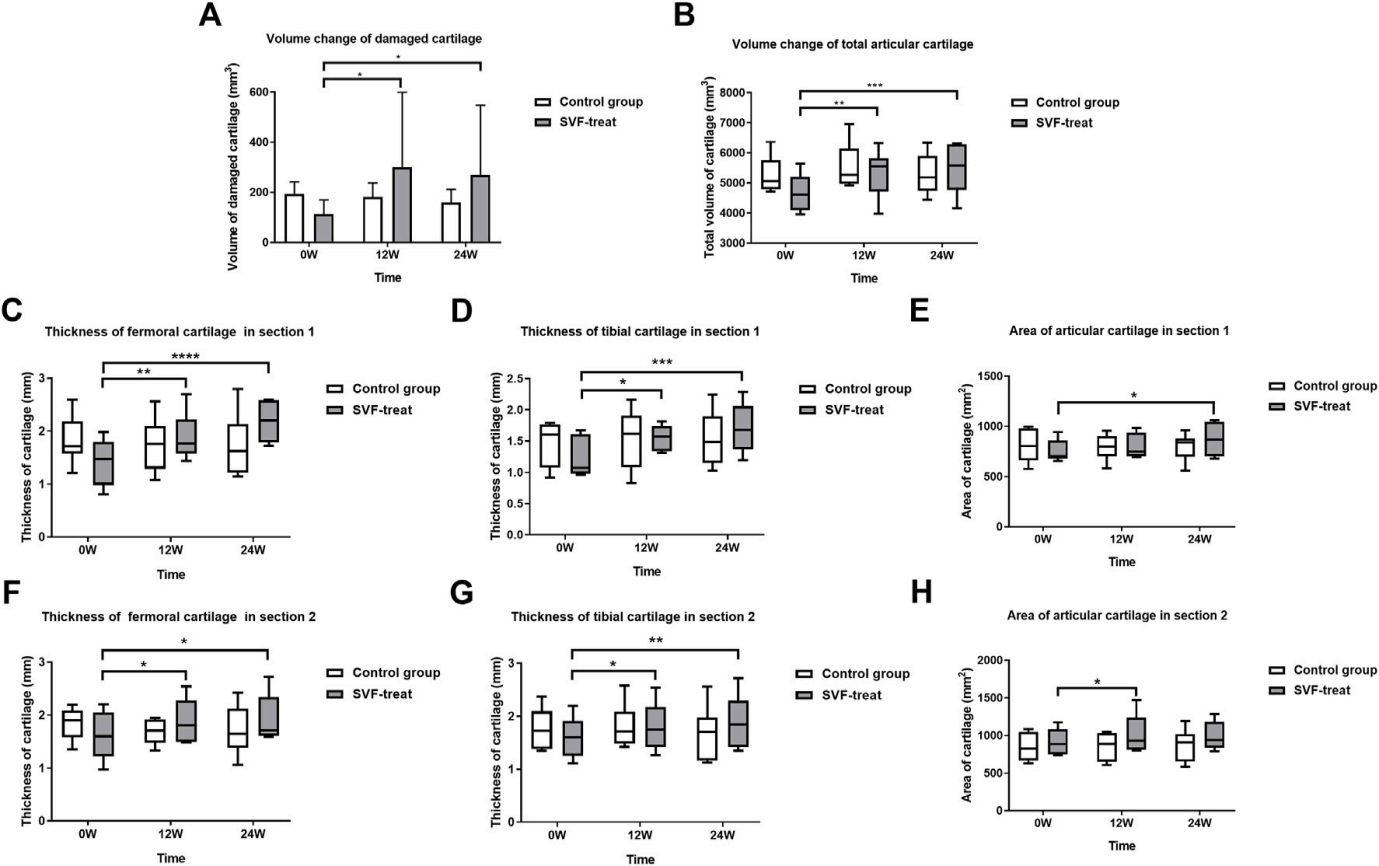
Radiologic evaluation of articular cartilage: volume, area and thickness. **(A)** Volume change of damaged cartilage. **(B)** Volume change of total articular cartilage. **(C)** Thickness of femoral cartilage in section 1. **(D)** Thickness of tibial cartilage in section 1. **(E)** Area of articular cartilage in section 1. **(F)** Thickness of femoral cartilage in section 2. **(G)** Thickness of tibial cartilage in section 2. **(H)** Area of articular cartilage in section 2. The volume of damaged cartilage and total cartilage, thickness and area of articular cartilage improved significantly in SVF treated group at 12 and 24 weeks as compared to baseline, while no significant difference was observed in control group.

We developed a novel method to provide 3D MRI imaging for human knee cartilage, enabling us to qualitatively evaluate the dynamic changes of cartilage defect in this study. As Figures 4, 5 demonstrate, after a single injection of SVF the femur and/or tibia cartilage defects of the treatment group show improvement, some show significant reduction at 12 and 24 weeks post therapy in comparison with pretreatment. In contrast, most the defects of the untreated group show deterioration and some new defects are noted.

### 3.5 Quantitative evaluation of cartilage value, area, and thickness demonstrated

By using femur or tibia as reference, the spatial alignment of knee cartilage 3D imaging of two different time points of the study from the same patient enable us to reliably and quantitatively evaluate the dynamic changes of cartilage volume, cross-sectional area, and thickness.

As illustrated in Figure 5, after a single injection of SVF in the treated knee joint, the mean cartilage volume of defect area increased from baseline 112.96 ± 56.85 to 301.42 ± 298.05 (*p* < 0.05) and to 279.58 ± 277.99 (*p* < 0.05) at 12 and 24 weeks post SVF therapy, respectively. No significant cartilage volume change was observed from week 12 to week 24. In contrast, the mean cartilage volume of defect area in the control group showed a decrease, trending from 210.54 ± 59.28 at baseline to 198.94 ± 71.22 (*p* > 0.05) at 12 weeks visit, and 179.64 ± 45.32 (*p* > 0.05) at 24 weeks follow-up. However, there was no statistical significance was detected.

The total volume of cartilage in SVF group increased from baseline (4668.61 ± 610.84) to 5335.21 ± 795.53 (*p* < 0.01) and 5483.52 ± 817.97 (*p* < 0.001) at 12 and 24 weeks post SVF therapy (Figure 5), respectively. By contrast, the total volume of cartilage in the control group was 5264.40 ± 613.30 at baseline, 5552.90 ± 784.41 (*p* > 0.5), and 5292.85 ± 698.96 (*p* > 0.5), at 12 and 24 weeks respectively. No significant difference was detected at each time point.

As Figure 5 illustrates, the thickness of femoral cartilage and tibia cartilage on both anterior and posterior coronal cross-sections were all increased significantly in comparison with that of baseline at 12 weeks (*p* < 0.5) and 24 weeks (*p* < 0.5) post therapy. The area of articular cartilage on both anterior and posterior coronal cross-sections were also increased in comparison with that of baseline at 12 weeks (*p* < 0.5) and 24 weeks, although at 24 weeks the increase was not statistically significant.

### 3.6 Changes in WOMAC index after SVF treatment

**FIGURE 6:**
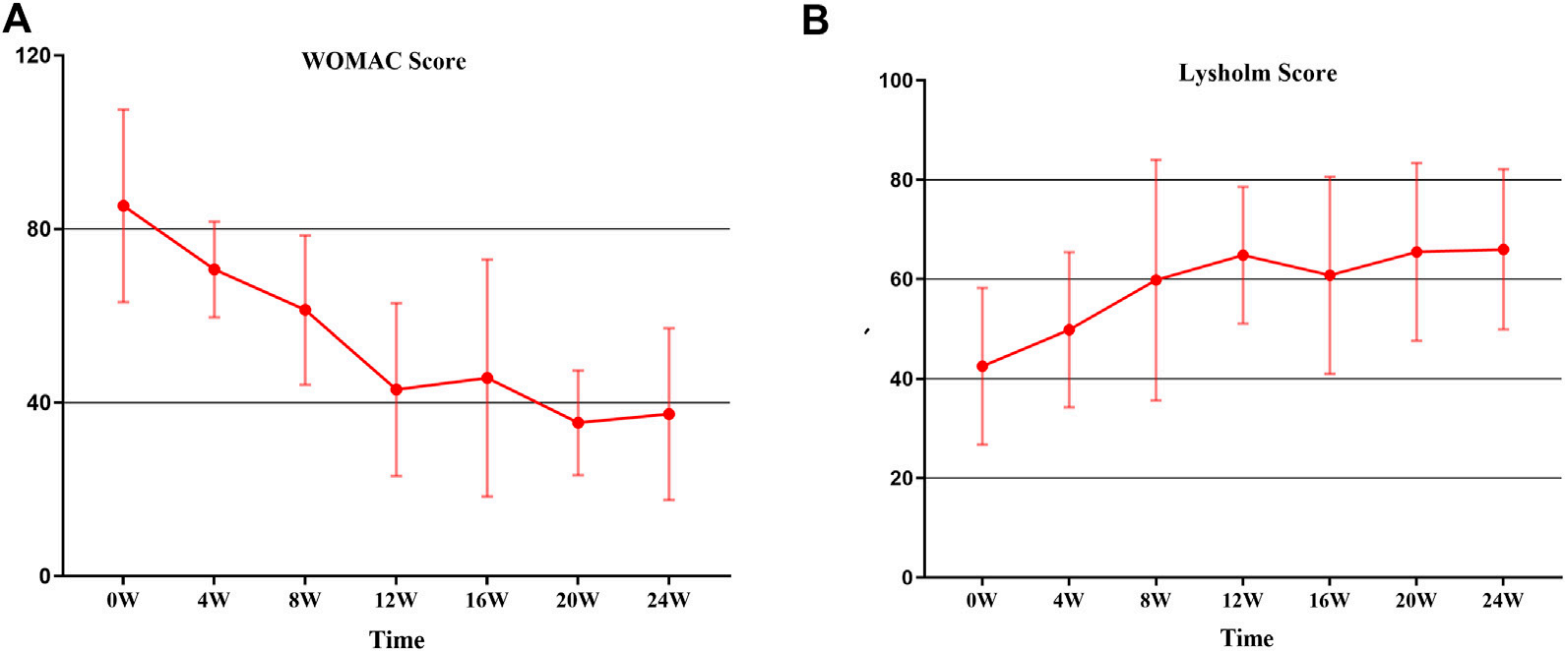
The trend in WOMAC score and Lysholm score from 0 to 24 W. **(A)** WOMAC Score: the decreasing trend of WOMAC is obvious between 0 and 12 weeks, and leveled off gradually at 12–24 weeks. **(B)** Lysholm score: The constant increase was observed from 0 to 12 weeks; thereafter, a relative stability was recorded.

The WOMAC score of 6 patients decreased from a preoperative mean of 85.33 ± 22.19 to a postoperative mean of 70.67 ± 11.02 at 4 weeks, 61.33 ± 17.61 at 8 weeks, 43.00 ± 19.93 at 12 weeks, 45.67 ± 27.32 at 16 weeks, 35.33 ± 12.10 at 20 weeks, and 37.33 ± 19.76 at 24 weeks, respectively (Figure 6A). Of note, the decreasing trend on the WOMAC score is obvious between 0 and 12 weeks, and levels off gradually at 12–24 weeks, indicating that the improvement of pain mainly occurred in the period of 0–12 weeks after SVF therapy.

### 3.7 Changes in Lysholm score after SVF treatment

On the basis of the Lysholm scale interpretation, a higher score represents better knee joint function. The results of 6 patients were that the Lysholm score increased from 42.50 ± 15.76 at the baseline to 64.83 ± 13.75 at 12 weeks; thereafter, a relative stability was recorded after 12 weeks (Figure 6B). The overall increase from the value before treatment to that at 12 weeks was found to be significant (*p* < 0.05). While similar to the WOMAC score, no statistically significant increase in the Lysholm score was detected after 12 weeks.

## 4 Discussion

Since 2011, Pak reported for the first time a series of knee OA cases treated with autologous adipose SVF (Pak, 2011), the clinical applications of SVF therapy for OA have attracted academic and clinical attention. Several studies or clinical observations on the application of SVF therapy for knee OA were reported (Koh and Choi, 2012; Pak et al., 2013; Koh et al., 2015; Pak et al., 2016b; Fodor and Paulseth, 2016; Tran et al., 2019). The safety and effects of relieving pain and improving function were observed in most patients. However, The lack of a quantitative measurement to provide convincing evidence of cartilage regeneration makes it impossible to obtain a strong conclusion on the correlation between clinical improvement and cartilage regeneration (Orozco et al., 2013; Koh et al., 2014; Orozco et al., 2014; Koh et al., 2015; Vega et al., 2015; Fodor and Paulseth, 2016). In addition, in some studies the inclusion criteria of the study subjects are K-L III-IV, indicating the progression of arthritis has entered a stage that requires surgical intervention (Orozco et al., 2013; Pak et al., 2016b; Pers et al., 2016; Sampson et al., 2016).

In this study we established a novel protocol to provide 3D MRI imaging for human knee cartilages and bones. The establishment of both cartilage and bone 3D MRI models of the knee joint is of crucial importance to achieve the goal to qualitatively and quantitatively evaluate cartilage regeneration. Since the knee articular cartilage is a thin layer of cartilage attached to the articular surface of femur and tibia, the articular surface of femur and tibia can be used as a reference to identify a thin bowl-shaped layer of cartilage. In addition, to achieve quantitative comparative analysis of the same patient’s articular cartilage MRI imaging data between two different periods (refer to MRI imaging data I and II), it is most critical to spatially align the cartilage section of MRI imaging data I and II in the same 3D spatial domain. Due to the minimum change of joint osteogenesis over a short period of time, and its well-defined anatomic structure, we spatially align the MRI imaging data I and II based on the osteogenic section to ensure that the cartilage section was registered in the same 3D spatial domain (Figures 1j–n). This ensure the cartilage volume; area and thickness of two different time periods are comparable. In this manner, the cartilage volume, area, and thickness of the same patient between two different time periods can be quantitatively compared (Figure 3).

Cartilage degeneration is commonly considered to be the initial pathological defect in OA. In this study, we hypothesized that SVF therapy facilitates and promotes cartilage regeneration and thus will have profound effects to control and reverse the cartilage degeneration in the OA patients with cartilage defect grades I/II, the early and mid stage of OA. In this pilot study six OA patients with both knee cartilage defect I-II were enrolled. The two knees of each patient were randomly assigned to autologous SVF (10^8^ cells) treatment group or non-treatment control group, thus this study minimized the potential impact of the differences of age, sex, BMI, life style, and medications between the treatment group and the control group on the interpretation of the study’s outcome. We estimated in this pilot study six patients were able to provide data for large scale clinical trails.

In this study, the adipose tissue harvest and SVF cells processing protocols were followed within the guidelines of the International Federation for Adipose Therapeutics (IFATS) and Science and the International Society for Cellular Therapy (ISCT) (Bourin et al., 2013). SVF products met releasing criteria, including cell viability test, endotoxin test, and bacteriologic test. In this study, we did not register any adverse events, confirming recent literature which reported that the SVF therapy for knee OA is a safe procedure (Pak et al., 2013; Koh et al., 2015; Fodor and Paulseth, 2016).

Similar to previous pilot studies, the WOMAC score decreased from a preoperative mean of 85.33 ± 22.19 to a postoperative mean of 43.00 ± 19.93 at 12 weeks and 37.33 ± 19.76 at 24 weeks, which represents decreasing pain and improving movement. Lysholm Knee Scale, another recommended method for measuring knee function, increased from 42.50 ± 15.76 at the baseline to 64.83 ± 13.75 at 12 weeks, and 66.10 ± 16.09 at 24 weeks. No statistically significant differences were detected at most time points in both the WOMAC score and Lysholm score which may be related to the limited sample size. The patient serving as self-control also affected the pain and function evaluation of SVF treated knee.

In this study, we qualitatively evaluated knee cartilage defects 3D MRI imaging. As illustrated in Figure 4 detailling that most defects of femur and/or tibia cartilage of the SVF treated group demonstrated improvement, some showed significant reduction at 12 and 24 weeks in comparison with 0 week. In contrast, most the defects of untreated group showed deterioration and some new defects were noted.

The quantitative evaluation of 3D MRI imaging of knee cartilage demonstrated that there was significant increase of cartilage value both in defect cartilage area and whole cartilage area of treated group (Figure 5). The cross-sectional area and thickness of the femoral and tibial cartilage at different positions were also significantly increased. This study shows that the defect cartilage achieved around 1.67 times improvement 12 weeks after SVF injection (from 112.96 ± 56.85 to 301.42 ± 298.05). Even in the cartilage non-defect area, the thickness and cross-sectional area were also improved to some extent. No obvious cartilage repair was observed from week 12 to week 24, which may indicate that the repair of cartilage by SVF therapy mainly occurred during the 12 weeks post treatment. In contrast, there was no significant change of cartilage value, thickness, and area within the untreated group (*p* > 0.5). Since only 6 patients were recruited in our clinical phase I/II study, a large-scale clinical phase III trial is required to further confirm the safety and efficacy of SVF therapy in treating chondral defects of knee osteoarthritis.

## 5 Conclusion

Our clinical phase I/II study indicated that SVF therapy is a safe clinical procedure for human knee OA. This study provided convincing evidence that SVF therapy reduced cartilage defects; significantly increased cartilage value, cross-sectional area and thickness, indicating SVF therapy facilitate cartilage regeneration in cartilage defect I-II knee OA patients 12–24 weeks post treatment. In this study, we have established a novel protocol to provide 3D MRI imaging for human knee cartilages and bones enabling us to qualitatively and quantitatively evaluate cartilage regeneration. The ZhongnanH articular cartilage defect index may provide a new tool to standardize diagnoses of cartilage defects of knee OA. This pilot study provided novel protocol and data for future large-scale phase III trial to further confirm the efficacy promises of SVF therapy.

From a public health point of view, if we are able to make early definite diagnoses for all knee OA patients, and provide safe and efficient therapy to facilitate cartilage regeneration, we will be able to control or reverse cartilage degeneration and completely change the epidemiology of OA worldwide. We hope our study will make a significant contribution to achieve this goal.

## Data Availability

The original contributions presented in the study are included in the article/supplementary material, further inquiries can be directed to the corresponding authors.
